# Non-Invasive Diagnostic Algorithm in Transthyretin Cardiac Amyloidosis: Is Bone Scintigraphy Always Enough?

**DOI:** 10.3390/jcm14238458

**Published:** 2025-11-28

**Authors:** Giulia Marchionni, Giulia Pecci, Maria Alfarano, Jacopo Costantino, Federico Ballatore, Federico Ciccarelli, Antonio Lattanzio, Nicola Galea, Giuseppe De Vincentis, Cristina Chimenti

**Affiliations:** 1 Department of Medical and Cardiovascular Sciences, Sapienza, University of Rome, 00185 Roma, Italy; 2 Department of Radiological, Oncological and Pathological Sciences, Sapienza, University of Rome, 00185 Roma, Italy

**Keywords:** cardiac amyloidosis, bone scintigraphy, cardiac magnetic resonance, cardiomyopathies

## Abstract

Technetium-99m bone scintigraphy has revolutionized the non-invasive diagnosis of transthyretin cardiac amyloidosis (ATTR-CM) and markedly increased disease recognition. Although this technique should ideally be applied in patients with a high pre-test probability of ATTR-CM, its use in other clinical settings may reveal incidental pathological myocardial uptake that prompts referral to specialized centers even in the absence of typical red flags. In such cases, where confounding factors such as left ventricular hypertrophy from alternative causes may coexist, awareness of potential pitfalls and the integration of scintigraphic findings with clinical features, biomarkers, and echocardiographic data are essential to avoid misdiagnosis and inappropriate therapy. Cardiac magnetic resonance (CMR) imaging provides a crucial complementary role, offering refined tissue characterization, improved differential diagnosis, and valuable prognostic insights. A combined approach that situates scintigraphy within the broader clinical context and incorporates CMR in ambiguous cases is fundamental to ensure an accurate diagnosis and optimal patient management.

## 1. Introduction

Transthyretin cardiac amyloidosis (ATTR-CM) is an infiltrative cardiomyopathy resulting from the myocardial deposition of misfolded insoluble protein fibrils [1]. Historically considered a rare and incurable disease, the landscape of ATTR-CM has been profoundly transformed over the last decade by significant advances in diagnostic and therapeutic approaches, irreversibly changing the epidemiological characteristics of this population [2]. In the past a definitive diagnosis of ATTR-CM required an endomyocardial biopsy (EMB) to identify amyloid deposits within the myocardium. However, advances in non-invasive imaging—particularly technetium-99m-labeled (99mTc) bone tracer scintigraphy—have revolutionized the diagnostic approach. A positive 99mTc-phosphate bone scintigraphy scan showing cardiac uptake, in the appropriate clinical context and after excluding light-chain amyloidosis, is now considered diagnostic for ATTR-CM without the need for a histological evaluation of the myocardial tissue [3,4,5]. This paradigm shift has greatly increased disease recognition and enabled the earlier initiation of disease-modifying therapies. Yet, alongside its high diagnostic yield, bone tracer scintigraphy has important limitations and potential pitfalls that must be understood to avoid false positives and, consequently, a misdiagnosis. This article will examine the role of 99mTc bone scintigraphy in the non-invasive diagnosis of ATTR-CM and discuss why this test should be trusted with caution in specific clinical scenarios. By synthesizing the existing body of evidence, from individual case reports to international guidelines, this review aims to provide a critical and up-to-date perspective on current approaches to the diagnosis of cardiac amyloidosis.

## 2. ATTR-CM: Diagnostic Algorithm and Early Detection

**Figure 1:**
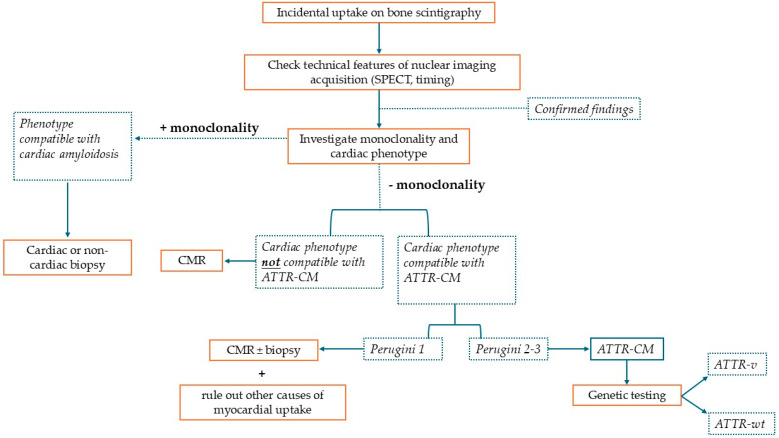
This is a figure representing the diagnostic algorithm that should be pursued when a patient presents with incidental myocardial uptake on bone scintigraphy performed for non-cardiac reasons. SPECT: single-photon emission computed tomography; ATTR-CM: transthyretin cardiac amyloidosis; CMR: cardiac magnetic resonance; ATTR-v: hereditary transthyretin amyloidosis; and ATTR-wt: wild-type transthyretin amyloidosis.

Cardiac scintigraphy with 99mTc-labeled bone-seeking tracers has emerged as a crucial tool for the non-invasive diagnosis of ATTR-CM. When a patient presents with unexplained increased left ventricular (LV) wall thickness (typically ≥ 12 mm) and clinical features suggestive of amyloidosis (Table 1), bone tracer scintigraphy can confirm ATTR-CM without a histological confirmation: a Perugini grade 2 or 3 uptake is considered diagnostic of ATTR-CM, alongside the exclusion of a monoclonal gammopathy [3,4,5]. This notion came from a prospective multi-center study including 1217 patients, in which bone scintigraphy using 99mTc-labeled bone tracers achieved a >99% sensitivity and an ~86% specificity for detecting ATTR-CM [6]. In this study, 72% of scans were performed using 99mTc-DPD, whole-body planar images were acquired 3 h after injection (except for some 99mTc-PYP images in which thoracic planar images were acquired 1 h after injection), and grading was performed through the Perugini scoring system. Most false positives in that series were due to patients having light-chain cardiac amyloidosis (AL-CA), which—once excluded—yields an extremely high specificity (~98–100%) for ATTR-CM to this imaging tool. These findings form the basis of the widely adopted non-invasive diagnostic algorithm proposed by Gillmore et al., in which a positive bone scan (grade ≥ 2) in the appropriate clinical context—plus a negative monoclonal workup—is sufficient to diagnose ATTR-CM without a biopsy. This algorithm has been endorsed by expert consensus and incorporated into recent guidelines, including the 2021 European Society of Cardiology (ESC) cardiac amyloidosis position statement and the ESC 2023 guidelines on cardiomyopathies [5,7]. The excellent diagnostic performance demonstrated in this prospective study was comparable to the one found in real-world settings: a recent meta-analysis reported a high diagnostic accuracy of planar visual grading, with an area under the curve (AUC) of 0.99, a sensitivity of 0.96, and a specificity of 0.96. In this study, including 23 studies for a total of 3954 patients who underwent bone scintigraphy, the quantitative analysis of SPECT imaging had a similar AUC (1.00, 95% CI 0.99–1.00) but a higher sensitivity (100%) and specificity (97%) [8]. These major developments in ATTR-CM diagnosis led to a greater ability to recognize this condition in its early stages [9]. On the other hand, this growing awareness has led to ATTR-CM suspicion arising from a positive result of bone scintigraphy used for different purposes [10]. This imaging method is, in fact, widely used also for oncologic follow-ups, especially for patients with prostate or breast cancer, in which bone involvement must be evaluated. In these patients, incidental myocardial uptake opens a parallel diagnostic pathway that prompts a referral to specialized centers. To date, an increasing number of patients are referred to cardiomyopathy referral centers for a myocardial uptake suggestive of cardiac amyloidosis as the presenting chief complaint, in most cases without a proper contextualization of the incidental finding [11]. In these patients, the diagnostic paradigm has shifted from looking for cardiac amyloidosis in a patient with systemic red flags to looking for systemic red flags in a patient with a bone scintigraphy diagnostic for ATTR-CM. In this context, an effort should be made to re-evaluate the whole clinical picture to support or exclude an ATTR-CM diagnosis. Sometimes, given the high prevalence of LVH and other potential biases, positive bone scintigraphy can coexist with additional findings supporting ATTR-CM, even in absence of cardiac amyloidosis, leading to inappropriate therapy. In this context, the critical integration of every element is of paramount importance, and cardiac MRI stands out as extraordinary tool to corroborate the diagnosis [12]. Figure 1 summarizes the desirable diagnostic pathway in a patient with incidental myocardial uptake on bone scintigraphy.

## 3. Typical Characteristics of Patients with ATTR-CM

The comprehensive interpretation of the entire clinical picture is universally recognized as the basis for the non-invasive diagnostic work-up for cardiac amyloidosis [3,5,7]. ATTR-CM typically presents in older adults, with the wild-type form most often manifesting in males in their 70s and 80s, while hereditary ATTR (ATTRv) may present earlier depending on the specific mutation and the phenotypic penetrance within the family [4]. Clinically, patients frequently report symptoms of congestive heart failure, especially exertional dyspnea, orthopnea, and lower limb edema, often in the context of a preserved ejection fraction [13]. Common history elements include atrial arrhythmias and conduction system disease at an early age and an intolerance to anti-hypertensive therapies that were previously well tolerated. In many patients, non-cardiac red flags precede or accompany cardiac symptoms: carpal tunnel syndrome, lumbar spinal stenosis, ruptured biceps tendon, unexplained peripheral neuropathy, or autonomic symptoms may be present at diagnosis [14]. The diagnostic delay is often considerable, as symptoms are insidious and may be misattributed to aging, hypertension, or other comorbidities such as diabetes or chronic kidney disease. The echocardiographic evaluation of patients with cardiac amyloidosis usually reveals concentric left ventricular hypertrophy, a small ventricular cavity, a reduction in tissue Doppler imaging velocities, elevated LV filling pressures, biatrial enlargement, and a reduction in global longitudinal strain (GLS) values with relative apical sparing [4,15,16,17]. Additional features include interatrial septum and valve thickening, right ventricular hypertrophy, and pericardial effusion. These characteristics are typical of full-blown ATTR-CM but might be absent in specific TTR mutations (i.e., Phe64Leu, Val30Met, and Ser77Tyr), non-ATTR forms, or early stages of the disease [3]. The same holds true for electrocardiographic findings, like low voltages, pseudo-infarct patterns in septal leads, conduction blocks, and atrial fibrillation, which are highly prevalent in this population but may be absent in a considerable percentage of patients [18,19,20,21]. Importantly, troponin T or I, and natriuretic peptides are commonly elevated in ATTR-CM, and they must be taken into consideration when evaluating this clinical context [22,23]. Systemic red flags represent a milestone of ATTR-CM diagnosis, but they might be absent or subtle in a minority of cases as well. Table 1 summarizes the clinical characteristics that should always be investigated in patients with ATTR-CM. As discussed in the next section, several conditions can produce false positive or false negative bone scans. Thus, the prudent and fundamental approach is to always integrate all anamnestic, clinical, and instrumental data to reach a proper diagnosis.

## 4. Myocardial Uptake: When and Why

Bone tracer scintigraphy has emerged as a pivotal imaging modality for the detection of cardiac amyloidosis because of its unique ability to bind selectively to amyloid fibrils within the myocardium [24]. Diphosphonates and pyrophosphates labeled with metastable technetium-99 have been recognized to accumulate in the myocardium as early as the 1980s. Their diagnostic utility for cardiac amyloidosis, however, was not established until the 2000s [25]. The mechanism by which bone tracers bind to the TTR amyloid is not yet fully understood: one prominent hypothesis suggests that the preferential binding in ATTR-CM is linked to the presence of microcalcifications within TTR deposits [26,27]. These microcalcifications have been described histologically in all ATTR-CM wild-type hearts and, to a lesser extent, in some AL-CA hearts. Another theory proposes that the tracer uptake is related to the different composition of the amyloid fibrils, with type A fibrils (associated with C-terminal protein fragments) appearing to bind 99mTc-DPD more readily than type B fibrils [28]. Several technetium-labeled tracers are recommended for identifying cardiac amyloid deposits: 99mTc-pyrophosphate (99mTc-PYP), 99mTc-3,3-diphosphono-1,2-propanodicarboxylic acid (99mTc-DPD), and 99mTc-hydroxymethylene-diphosphonate (99mTc-HMDP). While all three have demonstrated a comparable diagnostic performance in large cohorts, 99mTc-PYP is the only one approved in the United States, whereas 99mTc-DPD and 99mTc-HMDP are more commonly used in Europe [29]. A key difference lies in their extracardiac binding: 99mTc-PYP shows a very limited extracardiac uptake, while 99mTc-DPD and 99mTc-HMDP can reveal amyloid deposits in skeletal muscle, lungs, and soft tissues [30,31]. The Perugini grading system is commonly employed for the qualitative assessment of the cardiac uptake relative to bone uptake on planar images, ranging from grade 0 (no cardiac uptake) to grade 3 (significant cardiac uptake exceeding bone uptake), with a score of 1 indicating mild cardiac uptake (inferior to bone uptake) and a score of 2 indicating moderate cardiac uptake accompanied by attenuated bone uptake [25]. A Perugini score of grade 2 or 3, combined with the absence of monoclonal components in serum and urine, is diagnostic for ATTR-CM [7]. Quantitative methods such as the heart-to-contralateral lung (H/CL) ratio are also used, especially with 99mTc-PYP, where a ratio ≥1.5 at 1 h post-administration has a high sensitivity (97%) and specificity (100%) for differentiating ATTR-CM from AL-CA [32]. Commonly adopted thresholds are an H/CL ≥ 1.5 at 1 h and H/CL ≥ 1.3 at 3 h, reflecting the blood pool clearance that occurs over time [33]. Early imaging (1 h) may be preferable for a differential diagnosis, while delayed imaging (3 h) can better assess the disease extent and reduce false positives due to vascular activity. Differences in gamma camera systems, collimator designs, and energy window settings can introduce measurable variability (up to 10–15%) in planar and SPECT-based metrics. Therefore, cross-calibrations using standardized phantoms and the local validation of H/CL thresholds are recommended for multi-center reproducibility [34]. The need for the standardization of quantitative SPECT protocols has been emphasized in recent guidelines and review papers [32]. Recent studies have demonstrated that quantitative SPECT/CT with standardized uptake values (SUVs) can provide absolute tracer quantification, correcting for attenuation and scatter effects [35,36]. SUV-based indices (e.g., SUVmax, SUVmean) correlate well with Perugini visual grades and may outperform planar semiquantitative methods. This approach is particularly valuable when planar findings are borderline (grade 1), in longitudinal follow-ups, or in multi-center trials where harmonized quantitative metrics are required. However, its use should be limited to laboratories with validated calibration protocols and dedicated quantitative software.

## 5. Pitfalls and False Positives in Bone Tracer Scintigraphy

Though technetium-labeled cardiac scintigraphy has an excellent specificity for ATTR-CM in published studies, real-world experience has revealed important pitfalls and sources of false positive and false negative results [37]. Interpreting a bone tracer scan requires a careful technique and an awareness of conditions that can mimic myocardial radiotracer uptake, or else there is a risk of failing to detect the pathological accumulation of amyloids within myocardial cells. Of note, the absence of cardiac uptake in a clinical scenario suggestive of ATTR-CM does not rule out a diagnosis. Very early stages of cardiac infiltration may show a negligible uptake. Certain TTR variants, such as Phe64Leu, Ser77Tyr, Val30Met, Glu122Lys, and V122I, often present with mild or no uptake (Perugini grade 0–1), possibly due to conformational changes that mask tracer binding sites or a lower phenotypic expression in early stages [38,39,40,41]. Table 2 reports the predominant clinical phenotype associated with these variants and the proposed diagnostic approach. In these cases, if patients refuse the histologic assessment, follow-up scintigraphy might reveal a pathological uptake with disease progression.

Small case series reported false negative results also within the wild-type ATTR-CM cohort [42,43]. For this reason, in cases of a strong clinical suspicion for ATTR-CM, it is essential to proceed with a thorough diagnostic work-up that includes cardiac magnetic resonance imaging, genetic testing, and a comprehensive neurological assessment. Our current understanding of nuclear imaging findings in these subgroups has emerged through the use of endomyocardial biopsies, which represent the gold standard for confirming myocardial involvement in ATTR-CM: only the histological demonstration of amyloid deposition within myocardial tissue allows for an unequivocal diagnosis and enables the initiation of an appropriate disease-specific therapy in this setting [4]. In fact, it is fundamental to remember that the EMB retains a central role not only in patients with documented monoclonal gammopathy but also in all ambiguous scenarios in which non-invasive modalities yield discordant or equivocal results. In these clinical contexts, an EMB provides unmatched diagnostic specificity, allowing for differentiation between different amyloid precursor proteins as well as the exclusion of phenocopies and diagnostic certainty when the non-invasive assessment remains inconclusive. Because the amyloid infiltration of the myocardium typically exhibits a diffuse distribution, right ventricular sampling is generally adequate to achieve diagnostic accuracy, provided that multiple specimens are obtained; current recommendations suggest acquiring at least three tissue samples to minimize sampling errors and increase the diagnostic yield [44]. Following the confirmation of amyloid deposition by Congo red staining and the characteristic apple-green birefringence under polarized light, the precise identification of the amyloid precursor protein is essential for guiding treatment. Although immunohistochemistry and immunogold immunoelectron microscopy can assist in amyloid typing, these methods may be limited by antibody specificity and operator-dependent variability. In contrast, a mass spectrometry-based proteomic analysis remains the gold standard for tissue characterization, offering a robust diagnostic performance with a reported sensitivity of approximately 88% and a specificity of 96% [45]. Table 3 summarizes the principal clinical scenarios in which an endomyocardial biopsy becomes mandatory to establish a correct diagnosis and to ensure the timely implementation of targeted therapeutic strategies.

When speaking about false positive results, a plethora of possible scenarios must be considered: technical and physiological artifacts represent a considerable proportion of cases leading to misclassification. Among all possible factors, the one responsible for the most false positive scans is residual blood pool activity appearing as cardiac uptake on planar images. If the radiotracer remains in the cardiac blood pool (for example, due to slower clearance of Tc-PYP or suboptimal timing), the planar image may show diffuse cardiac region activity that is not true myocardial uptake [36]. Among patients with grade 2 uptake on planar images, follow-up Single-Photon Emission Computed Tomography (SPECT) imaging has shown a 64% false positive rate, mainly due to blood pooling or absent myocardial uptake [46]. For this reason, it is crucial to perform SPECT in addition to planar imaging for all positive or equivocal studies [3]. SPECT allows for three-dimensional localization and can distinguish the tracer in the myocardium versus activity in the cardiac chambers or adjacent structures [47]. Planar imaging alone is in fact no longer considered adequate for diagnosis, and current guidelines explicitly recommend SPECT (or SPECT/CT) to reduce misclassifications [4,7,11]. Other technical artifacts include the overlap of extracardiac uptake on the heart silhouette (a fractured rib or bone metastasis) that can concentrate the tracer and be projected over the heart on a planar view, mimicking myocardial uptake. Radiotracer biodistribution issues can also cause diagnostic errors: if the tracer is improperly prepared or in the presence of factors that can alter its distribution (e.g., in chronic kidney disease, bisphosphonate therapy, or recent intravenous iron administration), diffuse soft tissue uptake can occur, including the cardiac silhouette [48]. These situations, though fortunately rare, highlight the need for rigorous radiopharmaceutical quality control and careful scan interpretation by experienced readers. Box 1 reports the three fundamental aspects to keep in mind to avoid false negative results related to technical errors. Independently from the specific cause, if technical artifacts are suspected, bone scintigraphy with SPECT should be repeated as soon as possible.

Box 1 **Nuclear lab checklist.**

✓ Select proper timing for image acquisition
✓ Integrate SPECT in case of positive myocardial uptake
✓ Correlated hot spots with anatomy

Additionally, TTR is not the only amyloid precursor that can cause cardiac uptake on bone tracer scintigraphy. Up to 40% of AL-CA patients can exhibit variable degrees of cardiac uptake (Perugini grades 1–3) [49]. As previously stated, when a monoclonal gammopathy is detected, a reliance on non-invasive imaging alone is insufficient, and a tissue biopsy becomes compulsory [39]. The biopsy of an extracardiac site (such as the abdominal fat pad or salivary gland) may be pursued initially; however, if that sampling is inconclusive, progressing to an EMB is warranted as soon as possible. Early and accurate typing is crucial in the context of AL-CA, where survival and outcomes are highly dependent on the prompt initiation of targeted therapy [4]. Box 2 summarizes the clinical features that should raise suspicion for AL-CA.

Box 2 **Do not miss AL-CA checklist.**

✓ Monoclonal component
✓ Rapidly worsening HF
✓ Hypotension
✓ Severe neuropathy
✓ Nephrotic syndrome
✓ Refractory pleural and pericardial effusion
✓ Macroglossia

AL-CA: light-chain cardiac amyloidosis; HF: heart failure.

**Figure 2:**
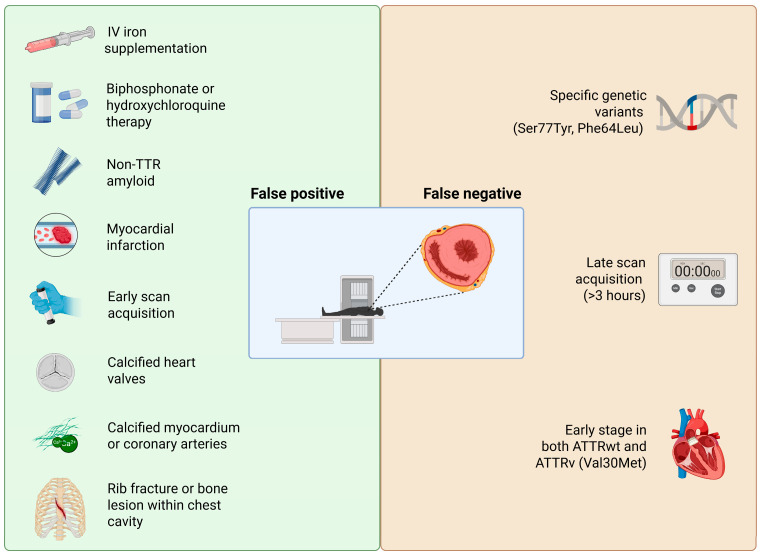
Factors that may lead to false positive or false negative results within the diagnostic pathway for ATTR-CM. Created in BioRender. https://BioRender.com/sakqah7 (accessed on 25 November 2025).

Rare amyloidosis types (AApoAI, AApoAII, AApoAIV, and Aβ2M) can also cause myocardial uptake [50,51]. These are exceedingly uncommon and typically require histology for diagnosis. Non-amyloid cardiac pathologies can cause false positive results as well: perhaps most intriguing are reports of bone tracer uptake in the heart due to conditions other than amyloids. Historically, technetium pyrophosphate was used in the 1970s for imaging acute myocardial infarction because it accumulates in calcium-rich necrotic tissue [52,53,54]. Accordingly, a recent myocardial infarction (especially within <1–2 months) can cause focal uptake in the infarct zone. These scenarios could theoretically lead to a false attribution of the uptake to amyloids if one is unaware of the recent infarct. Importantly, an old remote myocardial infarction scar usually does not take up the tracer (scar tissue lacks active calcium deposition), so prior infarcts >6 weeks old are less of a confounder. Another recognized pitfall is uptake due to extensive cardiac calcification. Bone-seeking tracers can bind to areas of calcium hydroxyapatite, so heavily calcified cardiac structures like dense mitral annular calcification can lead to significant uptake that could be misread as myocardial amyloid uptake [55]. Coronary arterial calcifications could potentially create a similar issue. While the blood vessels themselves are thin structures, extensive calcification in coronary plaques or in the myocardium (as can occur in end-stage renal disease with metastatic myocardial calcinosis) might generate focal hot spots of tracer uptake. Indeed, a case of diffuse myocardial calcinosis in the setting of non-ischemic cardiomyopathy was reported to mimic cardiac amyloidosis [56]. In general, any cause of increased radiotracer counts in or near the heart can yield a false positive result. This includes not only calcifications and infarcts but also certain cardiotoxic drugs or inflammatory processes. One notable example is hydroxychloroquine-induced cardiomyopathy: this rare toxic form of myocarditis can produce a hypertrophic phenotype with focal myocyte necrosis and has been associated with positive bone scintigraphy uptake in at least one report [57]. Figure 2 summarizes prevalent possible factors leading to false positive or false negative results using bone scintigraphy in ATTR-CM.

In practical terms, to avoid pitfalls the imaging physician should

- Exclude blood pool activity (delayed imaging and SPECT);
- Correlate any hot spots with the anatomy (to check for valvular or rib uptake);
- Verify that the uptake is diffuse in the myocardium rather than regional.

**Figure 3:**
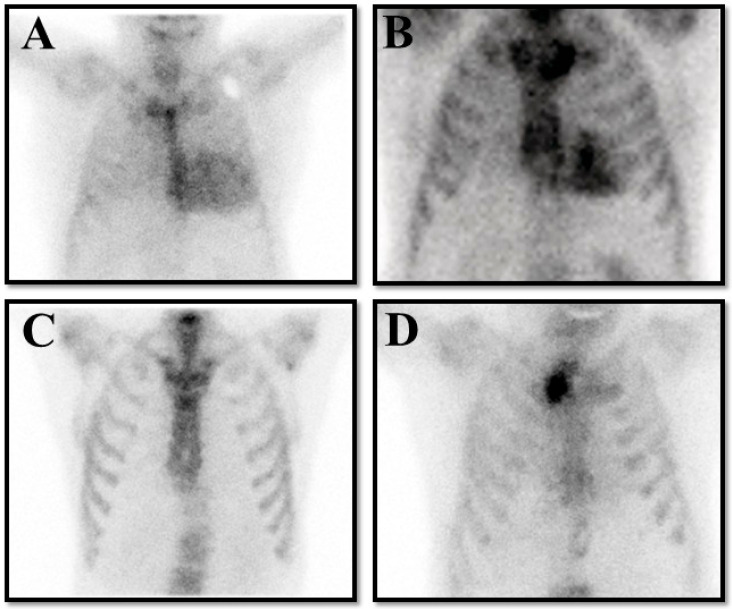
The panel represents four possible results on bone scintigraphy in patients with clinical suspicion of transthyretin cardiac amyloidosis (ATTR-CM). (**A**) is a true positive result compatible with Perugini grade 3. (**B**) is a false positive result in a patient with hypertrophic cardiomyopathy. (**C**) is a false negative result in a patient with hereditary ATTR-CM for a Val30Met pathological variant. (**D**) is a true negative result in a patient without ATTR-CM.

And the cardiologist should always integrate the resulting finding with clinical, electrocardiographic, and echocardiographic findings to ensure the patient’s clinical picture fits with ATTR-CM. When these precautions are taken, the specificity of a positive scan for true ATTR becomes unequivocal (Figure 3).

## 6. Role of Cardiac MRI in Differential Diagnosis

CMR imaging plays an invaluable complementary role in the work-up of suspected cardiac amyloidosis, particularly in complex cases or when scintigraphy results are ambiguous [3,4,7,11]. CMR offers high-resolution tissue characterization that can both support the diagnosis and help distinguish ATTR-CM from other causes of LV hypertrophy (Table 4).

Besides defining the myocardial morphology and function, CMR can directly visualize myocardial infiltration and fibrosis through late gadolinium enhancement (LGE) sequences and novel techniques like T1 mapping and extracellular volume (ECV) evaluations [58,59].

In cardiac amyloidosis (either ATTR-CM or AL-CA), CMR often shows a distinctive pattern of a diffuse global subendocardial LGE (involving the ventricles and sometimes atria) that may progress to a transmural LGE in advanced disease. One important distinctive feature of LGE sequences in cardiac amyloidosis is the altered gadolinium kinetics: in cardiac amyloidosis the rapid influx of gadolinium into the extracellular space, driven by the substantial amyloid burden, leads to myocardial nulling before or simultaneously with blood pool nulling, limiting the identification of the optimal T1 value that nulls the myocardium completely [60]. The myocardial T1 relaxation time and ECV are also markedly elevated due to the amyloid deposition. Native T1 mapping quantifies the longitudinal relaxation of tissue without the need for a contrast injection. Native T1 and T1 values after contrast injections allow clinicians to determine the ECV of the myocardium [61]. The ECV, derived from the partition coefficient corrected for the hematocrit, directly quantifies the extracellular space, and its highest values are found in patients with cardiac amyloidosis. CMR has a proven excellent diagnostic accuracy when compared with EMBs: in a large multi-center study including 160 patients with suspected cardiac amyloidosis undergoing both CMR and EMBs, LGE-based CMR demonstrated a sensitivity of 95% and a specificity of 98% for the diagnosis of cardiac involvement, while the combination of a characteristic LGE pattern and the absence of monoclonal proteins yielded a 98% specificity and a 99% positive predictive value for ATTR-CM [62]. A meta-analysis of seven studies further confirmed the high diagnostic value of CMR, showing a pooled sensitivity of 85.7% and a specificity of 92% against a histological confirmation [63]. Parametric mapping techniques, particularly native T1 and ECV quantification, have significantly enhanced diagnostic power: in comparative analyses, the ECV demonstrated a diagnostic odds ratio (DOR) of approximately 84.6, outperforming both the native T1 and LGE alone [64]. One study including 85 ATTR and 79 AL amyloidosis patients reported mean native T1 values of 1097 ± 43 ms for ATTR and 1130 ± 68 ms for AL, with an area under the curve (AUC) around 0.85 for distinguishing amyloidosis from other hypertrophic phenotypes [65]. Although specific LGE distribution patterns and wall thickness parameters can suggest the amyloid subtype—with one study reporting a sensitivity of 87% and a specificity 96% for ATTR identification [66]—other analyses indicate wide variability (sensitivity 28–99%, specificity 11–60%), highlighting that CMR alone cannot reliably differentiate between ATTR and AL cardiac amyloidosis using these parameters [67]. The native myocardial T1 and ECV correlate with functional indices, the LV mass, and prognostic biomarkers, with the ECV being the stronger indicator of prognosis. These imaging features, while not specific to the amyloid type, provide strong evidence of an infiltrative cardiomyopathy and can reinforce the diagnosis when nuclear scintigraphy is positive. Contemporary diagnostic algorithms consider typical CMR findings (global subendocardial LGE with abnormal T1/ECV) as a major clue or red flag for amyloidosis that should prompt confirmatory testing (such as a PYP scan or biopsy). Moreover, a reduction in the ECV has been linked to treatment responses in both ATTR and AL amyloidosis, demonstrating a greater accuracy in monitoring the disease progression and response to treatment than other imaging parameters [68,69]. In AL-CA patients, changes in the myocardial and liver ECV after 6 months from chemotherapy initiation independently predicted mortality [70]. Similarly, the ECV progression after one year was independently associated with an increased risk of mortality in patients treated with patisiran [71]. Unfortunately, despite being an exceptionally valuable modality for the evaluation of cardiac amyloidosis, cardiac magnetic resonance imaging still remains not universally accessible, as its implementation is often constrained by high costs, limited availability, and infrastructural disparities across healthcare systems.

## 7. Discussion

Technetium-99m bone scintigraphy has dramatically simplified the non-invasive diagnosis of ATTR-CM, increasing disease recognition, reducing the need for EMBs, and enabling the earlier initiation of disease-modifying therapies [10]. Nevertheless, as the clinical use of scintigraphy expands beyond cardiac amyloidosis settings, the awareness of its limitations becomes increasingly important. Incidental myocardial uptake in patients undergoing bone scans for non-cardiac reasons or pathological uptake due to alternative etiologies increase the risk of misinterpretation when not adequately contextualized within the overall clinical picture [24]. All potential pitfalls must therefore be considered. False positives can arise from residual blood pool activity, technical artifacts, myocardial infarction, extensive calcifications, AL-CA, or rare non-ATTR amyloid types. Likewise, false negatives may occur in early disease stages or in specific TTR variants. In these scenarios, the systematic integration of bone scintigraphy with biomarkers, electrocardiography, echocardiography, and clinical red flags is critical to avoid a misdiagnosis and inappropriate treatment decisions. Improperly labeling a patient with ATTR-CM means that the clinical attention may shift towards managing a disease the patient does not have, along with the prescription of drugs like Tafamidis that are unnecessary and also extremely expensive, while the true cause of the actual underlying condition remains unexplored or under-treated. In the same way, failing to treat a patient with cardiac amyloidosis because their scintigraphy results are non-diagnostic effectively condemns them to ongoing disease progression, with a profound impact on both prognosis and survival. CMR plays a crucial complementary role in this diagnostic landscape. By combining a high-resolution morphological assessment with advanced tissue characterization techniques, it refines the differential diagnosis of LVH and provides additional diagnostic certainty when scintigraphic findings are ambiguous [59]. This imaging technique occupies a pivotal role in the diagnosis and management of cardiac amyloidosis, owing to its unparalleled capacity for the non-invasive tissue characterization of myocardial involvement. Through advanced techniques such as LGE, native T1 mapping, and ECV quantification, CMR offers detailed insight into the interstitial infiltration, fibrosis, and myocardial architecture. Importantly, numerous studies have demonstrated that elevated ECV and T1 values are independent predictors of adverse outcomes in cardiac amyloidosis, underscoring its prognostic value [69,70]. Moreover, changes in myocardial tissue metrics—including reductions in the ECV—have been shown to correlate with the treatment response and survival [71,72]. This capability assumes growing importance in the current era, in which a rapidly expanding armamentarium of disease-modifying therapies for cardiac amyloidosis demands nuanced decisions about which patients should receive specific treatments, when a therapeutic class may need to be changed, and on what imaging- or biomarker-based criteria such decisions should rest. In this context, CMR emerges as a critical tool bridging diagnosis, risk stratification, and dynamic therapeutic monitoring, thereby enabling more precise patient selection, timely adjustments of therapy, and individualized management strategies. Differently, at this moment, changes in myocardial uptake on bone scintigraphy seem to poorly correlate with the response to treatment [73]. Larger studies are needed to investigate the correlation between changes in quantitative nuclear imaging metrics and responses to therapy.

## 8. Conclusions

Despite the remarkable simplicity and effectiveness of the current non-invasive diagnostic algorithm for cardiac amyloidosis, this review aims to encourage readers to adopt a consistently critical perspective when approaching a diagnosis. Such an attitude is essential to overcome the intrinsic limitations of bone scintigraphy and to avoid potential misclassification or inappropriate therapeutic decisions. In this clinical context, EMB remains an irreplaceable tool for excluding AL-CA and for confirming amyloid deposits when non-invasive tests yield ambiguous results. In this setting, CMR represents a decisive complementary modality, particularly in the presence of confounding factors that may limit the specificity of bone scintigraphy. When integrated into a comprehensive diagnostic pathway, CMR contributes not only to improving diagnostic accuracy but also to refining risk stratification and guiding therapeutic decisions. Ultimately, embracing a critical diagnostic approach combined with a multimodal imaging strategy strengthens diagnostic confidence, offers valuable prognostic insights, and enables the comprehensive assessment of treatment responses over time.

## 9. Future Directions

As the field of cardiac amyloidosis progresses, future research will focus on refining quantitative imaging biomarkers, like the ECV and native T_1_ values on CMR, as well as standardized uptake values (SUVs) or retention indices from bone scintigraphy and amyloid-specific Positron Emission Tomography (PET) tracers. To be used as surrogates for prognosis and the disease burden, they require rigorous validation across broader and more heterogeneous patient populations, including early-stage disease, different amyloid subtypes, and specific comorbidities. In parallel, computational methods including artificial intelligence and machine learning models are beginning to merge imaging parameters, biomarker profiles, genotypic data, and clinical indices to predict the disease subtype, trajectory, and optimal therapeutic strategy. These approaches hold considerable promise for moving toward imaging-based precision medicine in cardiac amyloidosis. Integrated deep phenotyping will allow for a greater understanding of the disease evolution, appropriate drug selection, and therapeutic monitoring, drastically improving patient care. Collectively, these developments have the potential to improve early detection, reduce misclassification, optimize patient management, and enhance outcomes in cardiac amyloidosis.

## Figures and Tables

**Table 1:** Clinical and instrumental finding suggestive for ATTR-CM.

Category	Red Flags
**Cardiovascular** **manifestations**	HF with preserved ejection fraction LVH not explained by abnormal loading conditions Intolerance to standard HF drugs (ACEi, ARNI, ARB, BB) Orthostatic hypotension or syncope Atypical angina with normal coronaries
**Extracardiac findings**	Bilateral carpal tunnel syndrome (often precedes cardiac involvement) Lumbar spinal stenosis Peripheral neuropathy Biceps tendon rupture Autonomic dysfunction (ortostatic hypotension, GI symptoms, erectile dysfunction)
**ECG**	Low QRS voltages despite LVH on echocardiography Pseudo-infarction pattern in septal leads (QS complex) Conduction disease (AV blocks) Atrial fibrillation
**Echocardiography**	Concentric LVH without dilation Severe diastolic dysfunction Left atrial enlargement and dysfunction Reduced longitudinal strain with apical sparing Right ventricular hypertrophy
**Cardiac MRI**	Diffuse subendocardial or transmural LGE Abnormal nulling of myocardium Elevated ECV
**Biomarkers**	Persistently elevated natriuretic peptides and troponins disproportionate to symptoms
**Monoclonal component**	Normal serum kappa/lambda FLC ratio (0.26–1.65) Absence of monoclonal protein in serum/urine IFE

HF: heart failure; ACEi: ace inhibitors; ARNI: angiotensin receptor–neprilysin inhibitor; ARB: angiotensin receptor blocker; BB: beta-blocker; LVH: left ventricular hypertrophy; AV: atrioventricular; LGE: late gadolinium enhancement; ECV: extracellular volume; FLC: free light chain; IFE: immunofixation electrophoresis; and AL-CA: light-chain cardiac amyloidosis.

**Table 2:** Clinical phenotype and diagnostic approach of TTR variants associated with false negative results in myocardial bone scintigraphy.

*TTR Variant*	Phenotype	Diagnostic Approach
*Val30Met*	Mixed (late onset) Predominantly neurologic (early onset)	CMR Neurological evaluation Cardiac/non-cardiac biopsy
*Phe64Leu*	Predominantly neurologic	CMR Neurological evaluation Cardiac/non-cardiac biopsy
*Ser77Tyr*	Predominantly neurologic	CMR Neurological evaluation Cardiac/non-cardiac biopsy
*V122I*	Predominantly cardiac	CMR Cardiac/non-cardiac biopsy
*Glu122Lys*	Predominantly cardiac	CMR Cardiac/non-cardiac biopsy
*Leu58His*	Mixed	CMR Neurological evaluation Cardiac/non-cardiac biopsy

**Table 3:** Clinical scenarios in which endomyocardial biopsy is indispensable to reach diagnosis.

Clinical Suspicion for ATTR-CM	Monoclonality	Scintigraphy	EMB
High ^1^	+	+ ^2^	Needed to exclude AL-CA; non-cardiac sites can be considered first
High ^1^	-	+ ^2^	Not needed except if dual pathology is suspected (i.e., HCM + ATTR-CM)
High ^1^	-	-	Needed to confirm clinical suspicion and to initiate appropriate therapy
Low	-	+ ^2^	Needed if nuclear imaging result appears to be accurate and the clinical picture is unclear
High ^1^	+	-	Needed to confirm AL-CA; non-cardiac sites can be considered first

^1^ High clinical suspicion is defined by presence of left ventricular hypertrophy with at least two red flags typical of ATTR-CM or in the presence of a pathogenic or likely pathogenic TTR variant. ^2^ Positive scintigraphy is defined as Perugini score of grade 2 or 3. EMB: endomyocardial biopsy. AL-CA: light-chain cardiac amyloidosis. HCM: hypertrophic cardiomyopathy. ATTR-CM: transthyretin cardiac amyloidosis.

**Table 4:** Common CMR features in different hypertrophic phenotypes.

	Cardiac MRI Findings			
	nT1 Values	ECV	LGE	Abnormal Myocardial Nulling
***Sarcomeric HCM***	↑	↑ colocalized with fibrosis	Patchy, mainly within hypertrophic segments	Absent
***Anderson–Fabry disease***	↓↓ (=/↑when replacement fibrosis develops)	= (↑ when fibrosis develops)	Inferolateral wall	Absent
***Cardiac amyloidosis***	↑↑↑	↑↑↑↑	Diffused subendocardial or transmural	Present
***Athletes’ heart***	=	=/low	/, Rarely junctional	/
***Hypertensive heart***	=	=	Rarely present in lone systemic hypertension	/

CMR: cardiac magnetic resonance; HCM: hypertrophic cardiomyopathy; nT1: native T1 values; ECV: extracellular volume; and LGE: late gadolinium enhancement.

## Data Availability

Data sharing is not applicable.

## Abbreviations

The following abbreviations are used in this manuscript:

ATTR-CM	Transthyretin cardiac amyloidosis
LV	Left ventricular
AL-CA	Light-chain cardiac amyloidosis
ATTRv	Hereditary transthyretin amyloidosis
GLS	Global longitudinal strain
TTR	Transthyretin
SPECT	Single-photon emission computed tomography
99mTc-PYP	99mTc-pyrophosphate
99mTc-DPD	99mTc-3,3-diphosphono-1,2-propanodicarboxylic acid
99mTc-HMDP	99mTc-hydroxymethylene-diphosphonate
CMR	Cardiac magnetic resonance
LGE	Late gadolinium enhancement
ECV	Extracellular volume fraction
